# In situ label-free imaging of hemicellulose in plant cell walls using stimulated Raman scattering microscopy

**DOI:** 10.1186/s13068-016-0669-9

**Published:** 2016-11-22

**Authors:** Yining Zeng, John M. Yarbrough, Ashutosh Mittal, Melvin P. Tucker, Todd B. Vinzant, Stephen R. Decker, Michael E. Himmel

**Affiliations:** 1 Biosciences Center, National Renewable Energy Laboratory, Golden, CO 80401 USA; 2 National Bioenergy Center, National Renewable Energy Laboratory, Golden, CO 80401 USA; 3 BioEnergy Science Center (BESC), Oak Ridge National Laboratory, PO Box 2008 MS6341, Oak Ridge, TN 37831 USA

**Keywords:** Hemicellulose, Xylan, Xylanase, Label-free imaging, Raman Spectroscopy, Stimulated Raman Scattering (SRS), Microscopy

## Abstract

**Background:**

Plant hemicellulose (largely xylan) is an excellent feedstock for renewable energy production and second only to cellulose in abundance. Beyond a source of fermentable sugars, xylan constitutes a critical polymer in the plant cell wall, where its precise role in wall assembly, maturation, and deconstruction remains primarily hypothetical. Effective detection of xylan, particularly by in situ imaging of xylan in the presence of other biopolymers, would provide critical information for tackling the challenges of understanding the assembly and enhancing the liberation of xylan from plant materials.

**Results:**

Raman-based imaging techniques, especially the highly sensitive stimulated Raman scattering (SRS) microscopy, have proven to be valuable tools for label-free imaging. However, due to the complex nature of plant materials, especially those same chemical groups shared between xylan and cellulose, the utility of specific Raman vibrational modes that are unique to xylan have been debated. Here, we report a novel approach based on combining spectroscopic analysis and chemical/enzymatic xylan removal from corn stover cell walls, to make progress in meeting this analytical challenge. We have identified several Raman peaks associated with xylan content in cell walls for label-free in situ imaging xylan in plant cell wall.

**Conclusion:**

We demonstrated that xylan can be resolved from cellulose and lignin in situ using enzymatic digestion and label-free SRS microscopy in both 2D and 3D. We believe that this novel approach can be used to map xylan in plant cell walls and that this ability will enhance our understanding of the role played by xylan in cell wall biosynthesis and deconstruction.

**Electronic supplementary material:**

The online version of this article (doi:10.1186/s13068-016-0669-9) contains supplementary material, which is available to authorized users.

## Background

Hemicellulose plays a critical role in plant cell wall assembly, maturation, and deconstruction, particularly in grass and secondary walls of woody plants. As an important resource biopolymer, xylan is widely available from various sources, such as energy crop biomass, where it represents 22% of corn stover and other grass agriculture residues [1]; and can be relatively easily extracted [2]. In the search for more efficient strategies to convert biomass to fuels and high-value chemicals, xylan represents unique challenges and opportunities. In the process of conversion of biomass to drop-in biofuels, xylan is both a significant sugar source for biofuels fermentation and yet, also a factor contributing to biomass recalcitrance. The fermentation of xylan also produces xylitol, a low-caloric sweetener and an agent against dental caries with huge marketability in the food industry and with clinical applications [3]. Xylo-oligosaccharides derived from xylan are widely used in food and health products as bioactive ingredients [4–10]. With proper cross-linking chemistries, xylan can form porous forms and gels [11]. The bio-derived and biocompatible foamy materials derived from xylan also have key roles in numerous non-fuel market sectors, including cosmetics, drug delivery, tissue engineering, insulation, and gas storage [12]. Sugar-based non-ionic surfactants produced from xylan have also attracted growing attention due to their biodegradability and excellent foaming properties [13]. With the advent of engineered biomaterials, such as cellulose nano-fibers, cellulose nanocrystals, chitosans, and polyvinyl alcohol polymers, xylan can be considered another polymer suitable for renewable and biodegradable films that are more economical than those made from oil-based polymers [14]. Because of numerous applications for xylan, extensive research has been conducted to develop chemical and biological approaches to effectively separate xylan from the rest of lignocellulose with high sugar yields. In the past, attempts have also been made to in situ modify the xylan in the plant cell wall to produce xylan derivatives [15–17]. Effective detection of xylan, particularly in situ imaging of xylan in the presence of other biopolymers, would provide new tools to study the assembly and liberation of xylan from plant materials.

Wet laboratory methodologies have been used in the past to provide accurate qualitative and quantitative measurements of carbohydrates. Depending on the nature of the samples under study, these analyses may involve specialized sample preparation steps to extract, hydrolyze, and remove moisture before chromatographic and/or spectroscopic analysis. Therefore, fast and non-destructive analytical techniques are needed. Infrared spectroscopy has been used to estimate the concentration of methoxyl and acetyl groups on xylan [18] and has been applied to corn stover, poplar, and spruce samples to analyze changes that are caused by various thermal chemical pretreatments [19, 20]. Near infrared spectroscopy combined with multivariate analytical statistical methods has also been used to assess the chemical composition of corn stover and switchgrass [21, 22] wood [23] and Kraft pulps [24]. In the past, we have also applied non-linear Raman microscopic techniques, such as coherent anti-Stokes Raman scattering (CARS) and stimulated Raman scattering (SRS) microscopies, to obtain high-resolution (~300 nm), chemically specific, and non-destructive imaging information regarding the distribution of important chemical compositions in the plant cell wall [25–29]. While the non-linear Raman techniques rely on the Raman activity of a specific chemical group, they provide signal intensity of orders of magnitude higher than spontaneous Raman microscopy, offering much faster imaging acquisition rates [26].

Raman spectra of plant cell walls contain the vibrational modes primarily from three major wall components: lignin, cellulose, and hemicellulose (mostly xylan). Besides some relatively weak peaks, lignin’s primary Raman contribution is located around 1600 cm^−1^. Cellulose has broad Raman contributions; however, the peaks around 1100 cm^−1^ are widely considered as the unique Raman peak for cellulose. In the past, 1100 and 1600 cm^−1^ have been chosen by most of the Raman imaging efforts to in situ map cellulose and lignin in plant cell walls [26, 30–33]. Conversely, in situ mapping of xylan using Raman imaging is not common. One key challenge is that xylan and cellulose have similar chemical groups. Cellulose is a linear homopolymer consisting of glucose residues linked together by β-1,4-glycosidic bonds. Due to the inter- and intra-chain hydrogen bonds, the structure of cellulose is highly ordered. There have been six different crystalline forms of cellulose reported: cellulose I, II, III_I_, III_II_, IV_I_, and IV_II_. Cellulose I and II are found in nature and the other allomorphs can be obtained by chemical or physical treatments [34]. Cellulose I is the main form of cellulose consisting of two allomorphs: cellulose Iα and Iβ, which differ by the hydrogen patterns (Additional file 1: Fig. S1). The inter-chain hydrogen bond *O*6-H–*O*3 is dominant in cellulose I, whereas in cellulose II, the *O*6-H–*O*2 is dominant. In cellulose I and II, the *O*3-H–*O*5 intra-chain hydrogen bonds make each cellulose chain rigid and linear in shape. In contrast, xylan in the plant cell wall has a variety of side chains attached to the linear β-1,4-xylopyranose backbone, forming an amorphous structure [35] (Additional file 1: Fig. S1). Besides the difference in the chemical composition of the backbone sugar units and the sidechains, cellulose and xylan have dramatically different organizations of chemical backbones, which could affect their Raman spectra. It has already been discovered that a Raman peak in the spectrum may contain contribution not only from a single chemical group, but from a composite of several interacting vibrational motions involving multiple sites in the molecule [36, 37]. A good example of this case is the Raman spectra from cellulose I and II: although they have exactly the same chemical groups and bonding, their different cellulose chain organizations lead to two different spectra [38].

Over the past few years, extensive effort has been devoted to distinguish Raman contributions of xylan from the rest of cell wall components; however, the results varied depending on the materials chosen for study and the separation methods used to extract the samples [39, 40]. By using extracted hemicelluloses from woody materials, hemicellulose was found to contribute broadly to the Raman spectrum, which made hemicellulose difficult to distinguish by Raman alone [39]. A combination of principle component analysis and multivariate curve resolution applied to the Raman spectroscopy of isolated cell wall polysaccharides suggested that the Raman peaks from hemicellulose overlapped with those of cellulose. However, in the cell wall, this result might be also explained by the possibility that hemicellulose is distributed homogenously with cellulose [41]. In flax fibers, it was suggested that the weak Raman peaks at 800–870 and 475–515 cm^−1^ can potentially be used to identify xylan [42]. Chu et al. reported that in Miscanthus the weak Raman peak at 478 cm^−1^ was related to the HCC and HCO bending of the C6 group in hemicellulose [43].

Due to the complex nature of the biomass substrate, separation of xylan from the rest of cell wall components seems to be inevitable for accurate xylan quantification. However, common chemical approaches create broad impacts for all the cell wall components. Here, we report an approach to use xylanases to specifically digest large portions of xylan in a carefully pretreated corn stover substrate. No detectable lignin or cellulose was digested by the assay. This approach allowed us to produce a series of artificial xylan “concentration ladders” in corn stover substrates. By comparing the Raman spectra from xylan model compounds obtained from different sources and with different degrees of polymerization, we determined several Raman bands that are sensitive to xylan and demonstrated that xylan in corn stover cell walls can be imaged at surprising detail.

## Results and discussion

### Raman spectral analysis reveals potential xylan-specific bands

**Fig. 1:**
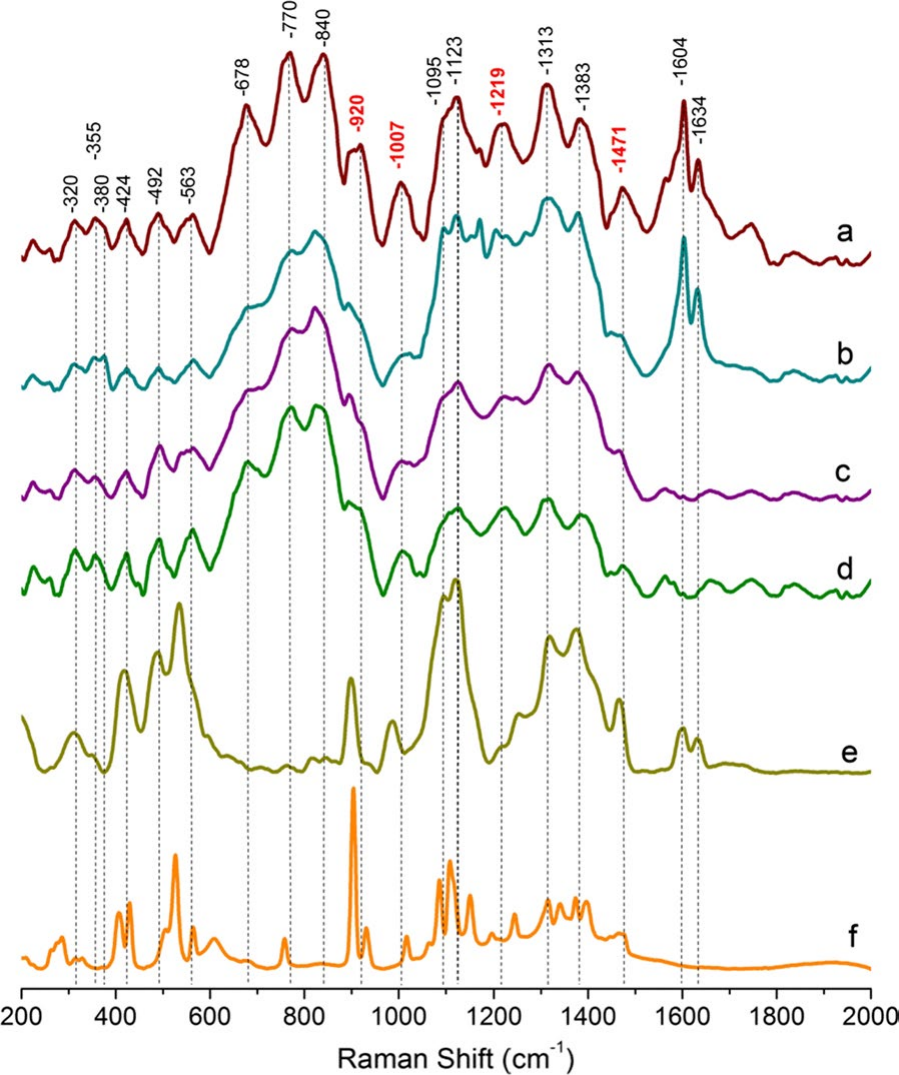
Comparison of normalized Raman spectra. *a* Deacetylated corn stover (control). *b* Deacetylated corn stover with 55% cell wall xylan removed by xylanases. *c* Oat spelts xylan. *d* Birchwood xylan. *e* Xylo-oligomer (DP ranging from 2–7). *f* d-xylose

Figure 1 (top row) shows the Raman spectrum from the undigested deacetylated corn stover control containing 31% of xylan, as well as cellulose and lignin (Table 1). After xylanase digestion, 55% of the cell wall xylan was removed (the resulting Raman spectrum is also shown in Fig. 1). The Raman spectra from the xylan model compounds obtained from various sources, with more than 68% xylan content (Table 1), are also listed in Fig. 1 for comparison. Those xylan model compounds have a wide range of degrees of polymerization (DP) from d-xylose monomer to short oligomers with DP 2-7; as well as extracted xylan polymers with DP100–200.

**Table 1:** Chemical compositional analysis (Wt%) of deacetylated corn stover control and extracted xylan models

Sample	Glucan %	Xylan %	Lignin %	Galactan %	Arabinan %	Fructan %	Mannan %	Acetate %	Total %
Deacetylated corn stover	45 ± 3	31 ± 2	13 ± 2	1.8 ± 0.5	1.7 ± 0.5	0.0 ± 0.1	0.0 ± 0.1	0.2 ± 0.1	93
Deacetylated corn stover with 55% cell wall xylan removed by xylanases	66 ± 4	12 ± 2	18 ± 2	1.0 ± 0.2	1.0 ± 0.2	0.3 ± 0.1	0.0 ± 0.1	0.1 ± 0.1	98
Xylo-oligo DP2-7	12 ± 2	73 ± 4	5 ± 2	3.4 ± 0.5	3.1 ± 0.5	0.0 ± 0.1	0.0 ± 0.1	1.6 ± 0.5	98
Oat spelts xylan	6 ± 1	68 ± 4	3 ± 1	1.0 ± 0.2	6 ± 1	0.0 ± 0.1	0.0 ± 0.1	5 ± 1	89
Birchwood xylan	1 ± 1	80 ± 5	2 ± 1	0.3 ± 0.1	0.5 ± 0.1	0.0 ± 0.1	0.00 ± 0.1	0.0 ± 0.1	84

Oat spelts and birchwood xylan samples also contain 3–4% and 10–12% glucuronic acid

Starting from the low frequency end, previous normal coordinate calculations revealed that the 250–600 cm^−1^ Raman region mainly contains the vibrational contributions from the skeleton bending involving CCC, COC, OCC, and OCO [44]. There are also small contributions from methine bending (CCH and OCH) and skeletal stretching (CC and CO) in this region [44]. Previous Raman spectroscopy studies of *Valonia* and Ramie cellulose found that the 320, 355, and 380 cm^−1^ peaks are associated with heavy atom bending [36, 44]. A Raman microscopy investigation of the tomato cell wall suggested that the 380 cm^−1^ peak belongs to δ(CCC) ring vibration from the cellulose [41]. Polarization Raman spectroscopy of the orientation of the chemical groups of cellulose indicated that the 320 cm^−1^ peak is associated with δ(CCC) ring vibration motions that are parallel to the cellulose chain axis [32], and that the 355 and 380 cm^−1^ peaks contain vibration motions both parallel and perpendicular to the cellulose chain axis [36, 37]. In the present study, after about 55% of cell wall xylan was hydrolyzed by enzymes, the 320, 355, and 380 cm^−1^ peaks showed very small changes. This observation indicates these three peaks are likely not directly linked to xylanase accessible xylan.

The 424, 492, and 563 cm^−1^ peaks appear in the deacetylated corn stover, the deacetylated corn stover following 55% xylan removal and the xylan model compounds. The 424 cm^−1^ peak was previously reported as δ(CCC) and δ(CCO) ring deformation in cellulose [38]. The 492 cm^−1^ peak is from cellulose glycosidic ν(COC) vibration [32, 42]. The 563 cm^−1^ peak was previously assigned to the cellulose δ(COC) ring vibration [38]. It seems, therefore, that the vibrations for 424, 492, and 563 cm^−1^ peaks could also exist in xylan. In fact, the Raman spectrum of the deacetylated corn stover sample with 55% xylan removed shows that these peaks are only slightly affected by xylanase digestion, indicating that they do contain some contribution from xylan. The 424, 492, and 563 cm^−1^ peaks also appear in the xylo-oligomers DP2-7, in which the 424 cm^−1^ and the 563 cm^−1^ peaks shift slightly to lower frequencies. In d-xylose, the 492 cm^−1^ glycosidic ν(COC) disappears, due to lack of any glycosidic bond and the 424 cm^−1^ ring deformation appears to degenerate into two peaks: the 407 cm^−1^ peak (ring bend coupled with OH bend) and the 433 cm^−1^ peak (ring deformation) [45]. It seems that in biomass, both cellulose and xylan contribute to the 424, 492, and 563 cm^−1^ bands. Enzymatic digestion of xylan alone from cell walls, therefore, should affect their peak intensity slightly.

The 678, 770, and 840 cm^−1^ peaks appear in the deacetylated corn stover control, the deacetylated corn stover sample with 55% xylan removed, oat spelts xylan, and birchwood xylan. Previously, it was reported that the 678 cm^−1^ peak was due to the low-frequency vibrations of the pyranoid rings [46]. In deacetylated corn stovers (Fig. 1a, b), the 770 cm^−1^ peak could be related to xyloglucan [47], and possibly some contribution from lignin aromatic rings, their substituent groups and side chains [39, 48] or ferulic acid attached to xylan. In oat spelts and birchwood (Fig. 1c, d), the 770 cm^−1^ band could be due to glucuronic acid group attached to xylan [49]. The Raman bands in the 830–900 cm^−1^ region have been related to the anomeric configuration of carbohydrates [46, 50–52].

The broad peak centered at 920 and the 1007 cm^−1^ peak found in the deacetylated corn stover control also appear in all of the xylan model compounds. For example, in d-xylose, there is a very strong peak at 900 cm^−1^ (symmetric COC str coupled with CC ring str) and a weak side peak at 930 cm^−1^ (the CO and the CC stretch coupled with methine deformation) [45]. The 900 cm^−1^ peak remains strong in the xylooligmers (DP2-7). In the oat spelts xylan, birchwood xylan, and deacetylated corn stover samples, this peak could be slightly overlapped with the lignin peak at 920–930 cm^−1^ (lignin skeletal deformation of aromatic rings, substituent groups, side chains, and CCH wag) [53]. The cellulose COC symmetric stretch also contributes to the 900 cm^−1^ [32]. After xylan removal, the 920 cm^−1^ peak is significantly reduced. It seems that this peak is associated with xylan, possibly COC stretch coupled with CC ring stretch. The d-xylose also has a medium strong peak at 1015 cm^−1^ due to CC and CO ring stretch coupled with methine deformation [45]. All the other xylan model polymers show a peak around 1007 cm^−1^ due to the ν(C–C) and ν(C–OH) vibrations [40]. The 1007 cm^−1^ in deacetylated corn stover is significantly reduced when xylan is digested, indicating that it is associated with xylan, possibly via the ν(C–C) and ν(C–OH).

Both the 1095 and 1123 cm^−1^ peaks have traditionally been assigned to cellulose for its COC stretch and COC stretch plus ring breathing, respectively. It is known that xylan and glucomannan have a very small contribution to these peaks [39]. In the past, the 1095 and 1123 cm^−1^ peaks have been widely used to image cellulose content in the cell wall [25, 26, 30–32, 54]. As shown in Fig. 1, both of the peaks showed little change after enzymatic digestion of the xylan, indicating that the enzyme has minimal impact on cellulose.

The 1219 cm^−1^ peak in the deacetylated corn stover control also shows up in all the xylan model compounds. In d-xylose, the 1219 cm^−1^ peak shifts to 1246 cm^−1^, which has been reported as the methine deformation coupled with OH in-plane bend in d-xylose [45]. In the xylo-oligomer (DP2-7), it appears as a similar peak around 1246 cm^−1^, possibly due to the C-O stretch, δ(CH) and/or δ(COH) in hemicellulose [39, 41, 55–57]. Some acetyl groups present on hemicellulose were also believed to contribute to the 1250 cm^−1^ peak [57, 58]. The 1219 cm^−1^ peak is greatly affected when the cell wall xylan is solubilized by enzymes, confirming that this peak is closely related to xylan, possibly associated with the COH and CH groups of xylan.

The δ(CH) and δ(COH) vibrations of xylan contribute to the 1313 cm^−1^ found in the deacetylated corn stover control [39, 40]. Some studies have specifically assigned the 1313 cm^−1^ peak to the C3–OH vibration on xylan [40]. In d-xylose, the 1313 cm^−1^ peak is associated with anomeric methine deformation coupled with OH in-plane bend, and the 1340 cm^−1^ peak is associated with methine deformation coupled with methylene wag [45]. Besides xylan however, cellulose also contributes significantly to 1318-1335 cm^−1^ by ω(CH_2_), δ(HCC), δ(HCO), and δ(COH) vibrations [32, 38]. It has been reported that lignin also contributes in the region 1297–1334 cm^−1^, possibly due to its aliphatic O–H bend vibration [39, 48] or vibration modes from S lignin [33]. The 1313 cm^−1^ peak is only partially reduced when xylan is enzymatically digested, confirming that it has partial contribution from xylan. The 1383 cm^−1^ peak in xylan could be due to the δ(CH) and δ(OH) vibration which were previously recorded at 1376 cm^−1^ [40]. In the deacetylated corn stover, oat spelts xylan, and birchwood xylan samples studied, it is likely that the 1383 cm^−1^ peak is also partly overlapped with the lignin peaks 1363 cm^−1^ and 1393 cm^−1^ from the C–H bend in the R_3_C–H and the phenolic O–H bend in the lignin, respectively [39, 53]. Besides this, cellulose also contributes strongly to 1380 cm^−1^ with its δ(CH2), δ(HCC), δ(HCO), and δ(COH) vibrations [38, 39]. The 1383 cm^−1^ peak thus has little diagnostic value. The 1313 cm^−1^ peak is only partially affected by enzymatic xylan removal, indicating that it involves only partial Raman contributions from xylan.

The 1471 cm^−1^ peak was found in deacetylated corn stover samples and in all the xylan models including the monomer d-xylose. Previously, Raman studies on xylose have shown that the peak 1471 cm^−1^ is due to the OH in-plane bend coupled with the methine deformation (1455 cm^−1^) and the methylene scissors coupled with the wag vibration (1480 cm^−1^) [45]. In the Raman studies of black spruce, the 1471 cm^−1^ peak was also considered for xylan [39]. By comparing the Raman spectrum of deacetylated corn stover before and after enzymatic xylan removal, the 1471 cm^−1^ peak exhibits significant drop when the cell wall xylan content is solubilized, indicating that it is highly associated with xylan and possibly related to the OH and CH_2_ vibrations in xylan.

The two peaks at 1604 and 1634 cm^−1^ are the signature peaks known for lignin. Specifically, the 1604 cm^−1^ peak is due to the aromatic ring vibration, and the 1634 cm^−1^ is assigned to the C=C stretch in the coniferaldehyde unit of lignin [59–62]. As shown in Fig. 1, both of the peaks showed little change after xylan digestion, indicating that the enzyme digestion has minimal impact on lignin.

### Xylan-content cell wall ladder confirms xylan-specific Raman bands

In the above discussions (also summarized in Table 2), it was found that the Raman bands at 920, 1007, 1219, and 1471 cm^−1^ are sensitive to cell wall xylan. To verify their dependence on xylan content, a series of deacetylated corn stover cell wall samples with relative xylan contents ranging from 45 to 100% (100% for native cell wall xylan content) was created (Additional file 1: Table S1, S2). No lignin or cellulose solubilization was detected by compositional analysis (Additional file 1: Table S1).

**Table 2:** Summary of Raman bands assignments

Deacetylated corn stover				Xylan models					
Control	After 55% xylan removal	Assignment	Species	Oat spelts xylan	Birchwood xylan	Xylo oligo DP2-7	Assignment	d-Xylose	Assignment in d-xylose
320w	320w	δ(CCC) ring	Cellulose	320w	320w	320m			
								330vw	OH bend coupled with CO def
355w	355w	δ(CCC) ring	Cellulose	355w	355w	355sh		–	
380sh	380w	δ(CCC) ring	Cellulose	–	–	–		–	
								407s	Ring bend coupled with OH bend
424m	424m	δ(CCC) and δ(CCO) ring	Cellulose, xylan	424m	424m	415s	Xylan δ(CCC) and δ(CCO) ring	433s	CO deformation
492m	492m	Glycosidic ν(CCC)	Cellulose, xylan	492m	492m	492m	Xylan glycosidic ν(CCC)	–	
530sh				530sh	530sh	530s		530s	CO deformation couple with ring bend
563m	563m	δ(COC) ring	Cellulose, xylan	563m	563s	570sh	Xylan δ(COC) ring	567m	CO def coupled with methine def
								613m	CO def coupled with CO ring bend
678s	678sh	Pyranoid rings		678sh	678m	–		–	
770s	770m	Xyloglucan, possibly some lignin ring and side chains	Xyloglucan lignin, ferulic acid	770m	770s	–	Glucuronic acid remainings	760s	CO ring str and bend coupled with CC ring str and CO str and bend
820–860s	820–860s	Glycosidic bonds and backbone vibration in hexose		820-860s	820-860s	–		–	
920m	920sh	COC str coupled with CC ring str.	Xylan	920sh	920sh	900s	Xylan COC str coupled with CC ring str	907vs	Symmetric COC str coupled with CC ring str
								930m	CO and CC stretch coupled with methine deformation
1007s	1007w	ν(C–C) and ν(C–OH)	Xylan	1007w	1007m	990m	Xylan ν(C–C) and ν(C–OH)	1019m	CC and CO ring str coupled with methine deformation
1095m	1095m	COC stretch symmetric	Cellulose	1095sh	1095sh	1095sh		1089s	CO str coupled with CC str and methine def
1123s	1123s	ν (COC), glycosidic; ring breathing, symmetric	Cellulose	1123m	1123m	1123m		1111s	CO str coupled with CC ring str
1219s	1219w	C-O str, δ(CH) and/or δ(COH)	Xylan	1219m	1219s	1250w	C–O str, δ(CH) and/or δ(COH)	1240m	Methine def coupled with OH i.p. bend
1313s	1313m	Cellulose aliphatic O–H bend vibration, xylan C3-OH	Cellulose, xylan	1313m	1313s	1315m		1313m	Anomeric methine def coupled with methine def
								1340m	Methine def coupled with methylene wag
1383m	1383m	Cellulose δ(CH2), δ(HCC), δ(HCO) and δ(COH), lignin phenolic O–H, xylan δ(CH) and δ(OH)	Cellulose, lignin, xylan	1383m	1383m	1383s	Xylan δ(CH) and δ(OH)	1383m	Methylene wag coupled with OH i.p. bend and methine def
								1395m	OH i.p. bend coupled methylene wag and methine def
1471s	1471w	Xylan OH and CH2	Xylan	1471w	1471m	1471s	Xylan OH and CH2	1471m	Methylene scissors coupled with wag
1604s	1604s	Lignin aromatic ring	Lignin	–	–	1604m	Residual lignin aromatic ring and coniferaldehyde	–	
1634s	1634s	C=C stretch in coniferaldehyde lignin	Lignin	–	–	1634m		–	

Conventional symbolism indicating relative intensity

*vw* very weak, *w* weak, *m* medium, *s* strong, *vs* very strong, *sh* shoulder

**Fig. 2:**
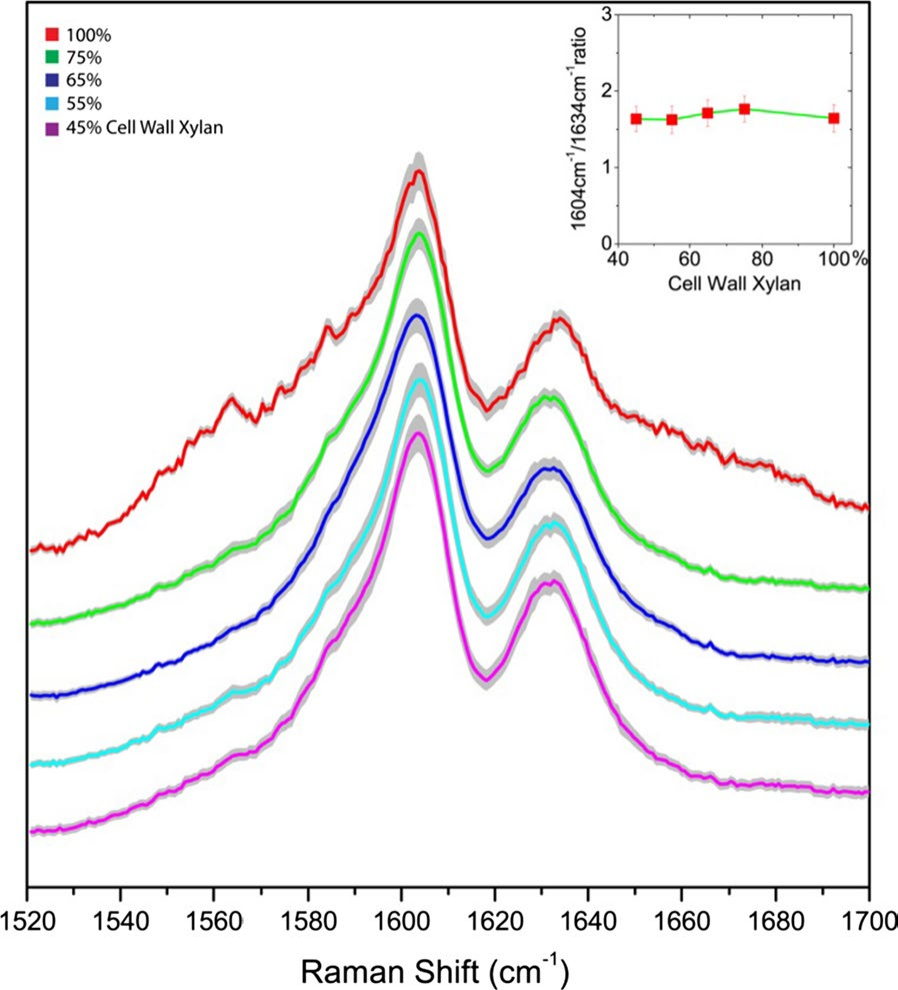
Comparison of lignin’s signature Raman peaks at 1604 and 1634 cm^−1^ in deacetylated corn stover cell walls with varying amounts of xylan content due to controlled xylanase digestion of xylan. The *gray traces* indicate the standard deviations. The two lignin signature Raman bands are unaltered even upon removal of significant amounts of cell wall xylan. The *inset* shows the peak ratio of 1604/1634 cm^−1^, which remains constant across wide ranges of xylan digestion. All spectrums are normalized

Figure 2 shows the comparison of the lignin-specific Raman band at 1600 cm^−1^ from the deacetylated corn stover cell wall samples with various xylan contents. The error bars in each spectrum show the standard deviation of hundreds of spectrum scans. All the spectra are normalized by the 1604 cm^−1^ peak for comparison (baseline leveled at 1520 cm^−1^). Although the xylan content varies significantly, the lignin peaks are almost identical in the range from 1520 to 1700 cm^−1^. The peak height of the 1634 cm^−1^ peak is almost identical for both the control and the enzyme-digested deacetylated corn stover. The peak ratio of 1604 to 1634 cm^−1^ (Fig. 2, inset), reflecting relative contributions from the aromatic ring vibration versus the C=C stretch in the coniferaldehyde unit of lignin, is constant for all the xylan concentrations. This implies that the ratio of coniferyl units and aromatic rings is constant.

**Fig. 3:**
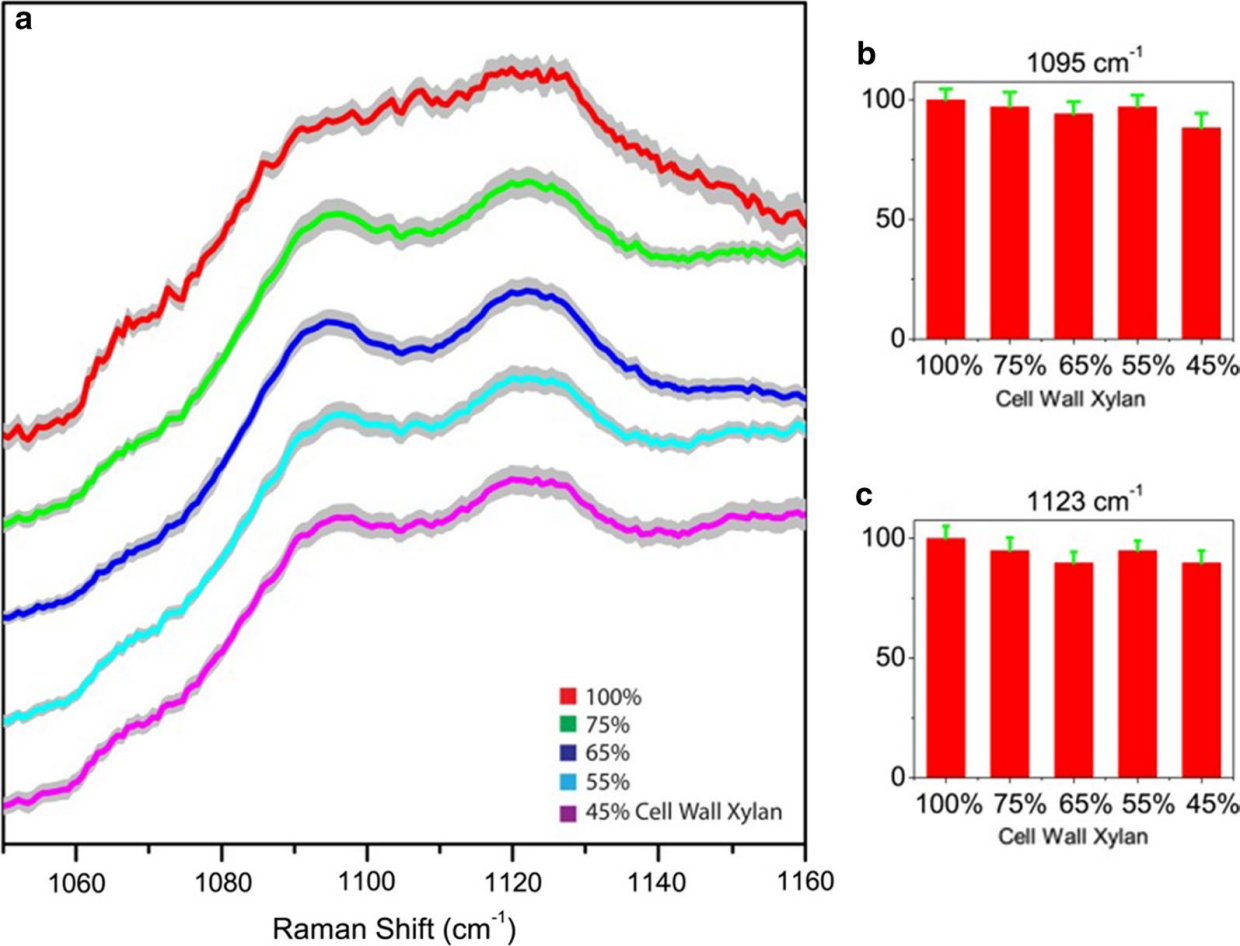
Comparison of cellulose’s signature Raman peaks at 1095 and 1123 cm^−1^ in deacetylated corn stover cell walls with varying amounts of xylan content due to controlled xylanase digestion of xylan. **a** Raman spectra show little change. **b, c** The peak heights of 1095 and 1123 cm^−1^ in cell walls remain constant with varying amounts of cell wall xylan content. All spectrums are normalized. The *gray traces* indicate the standard deviations

Figure 3 shows the comparison of cellulose-specific Raman bands from the deacetylated corn stover control and the cell wall samples with altered xylan contents. For comparison, these spectra are normalized by the lignin peak at 1604 cm^−1^. The two cellulose signature peaks, 1095  and 1123 cm^−1^, show similar shape for all the samples, albeit with significantly different xylan content (Fig. 3a). The peak heights of 1095 and 1123 cm^−1^ (baseline at 1050 cm^−1^) remain the same for the control and the enzyme-digested deacetylated corn stover cell wall (Fig. 3b, c). This result is an indication that the cellulose content in the cell wall is not affected by the xylanase treatments used for these samples.

**Fig. 4:**
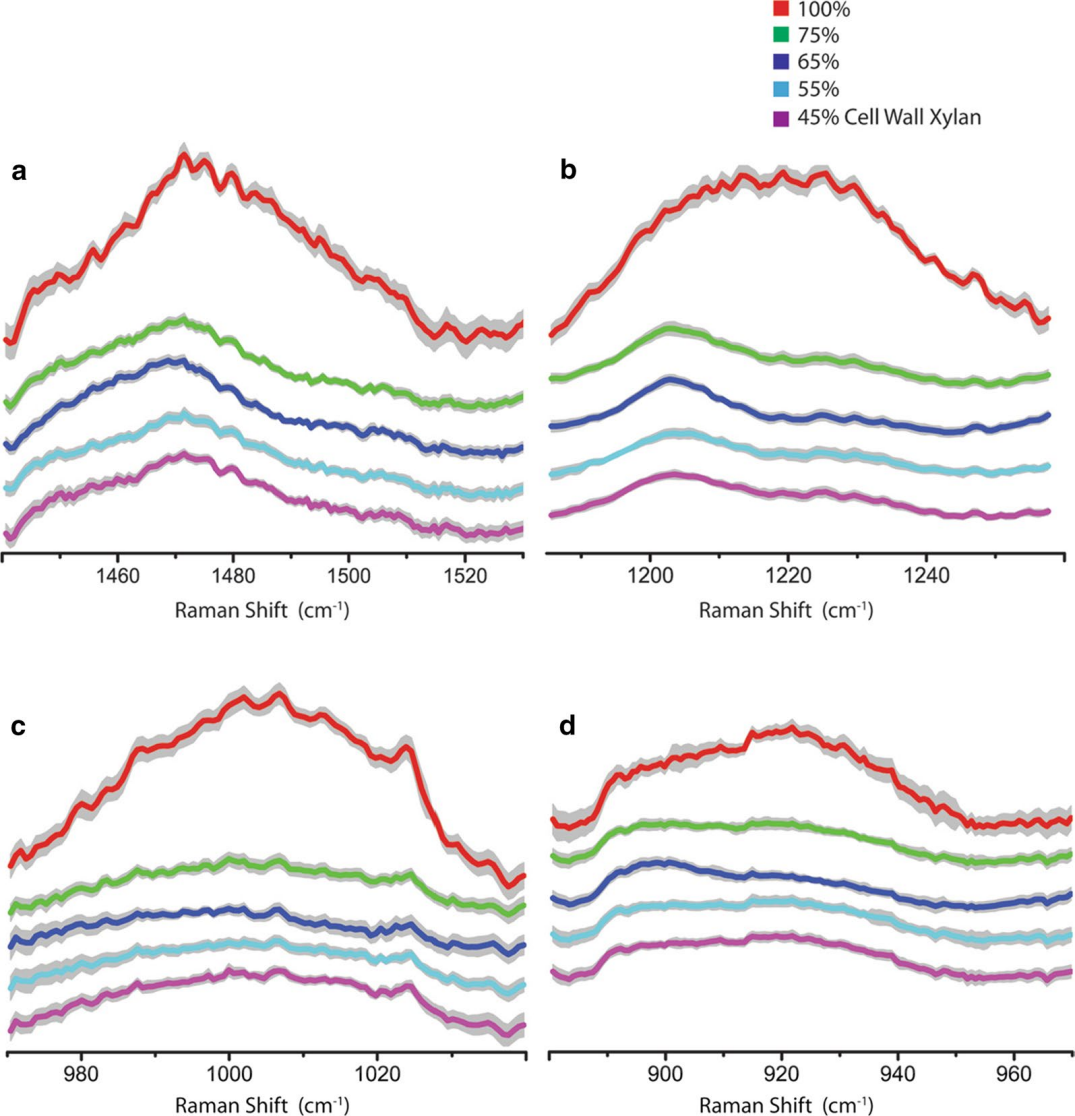
Zoom-in comparisons of the xylan-specific Raman bands with varying amounts of xylan content due to controlled xylanase digestion of xylan. **a** 1471 cm^−1^ (xylan OH and CH_2_), **b** 1219 cm^−1^ (xylan C-O str, δ(CH), δ(COH)), **c** 1007 cm^−1^ (xylan ν(C–C) and ν(C–OH)) and **d** 920 cm^−1^ (xylan COC str coupled with CC ring str) in deacetylated corn stover cell wall with varying amounts of xylan content due to controlled xylanase digestion of xylan. The *gray traces* indicate the standard deviations. All spectrums are normalized

In contrast to the cellulose and lignin bands, the four xylan-sensitive bands at 920, 1007, 1219, and 1471 cm^−1^ exhibit a dramatic and consistent reduction for all four of the xylan-removed cell walls samples (Fig. 4). The changes in the spectra of the xylan-removed cell walls are significantly greater than experimental error, indicating that those Raman bands are indeed associated with xylan removal.

### Xylan-specific Raman bands for in situ xylan imaging by SRS microscopy

**Fig. 5:**
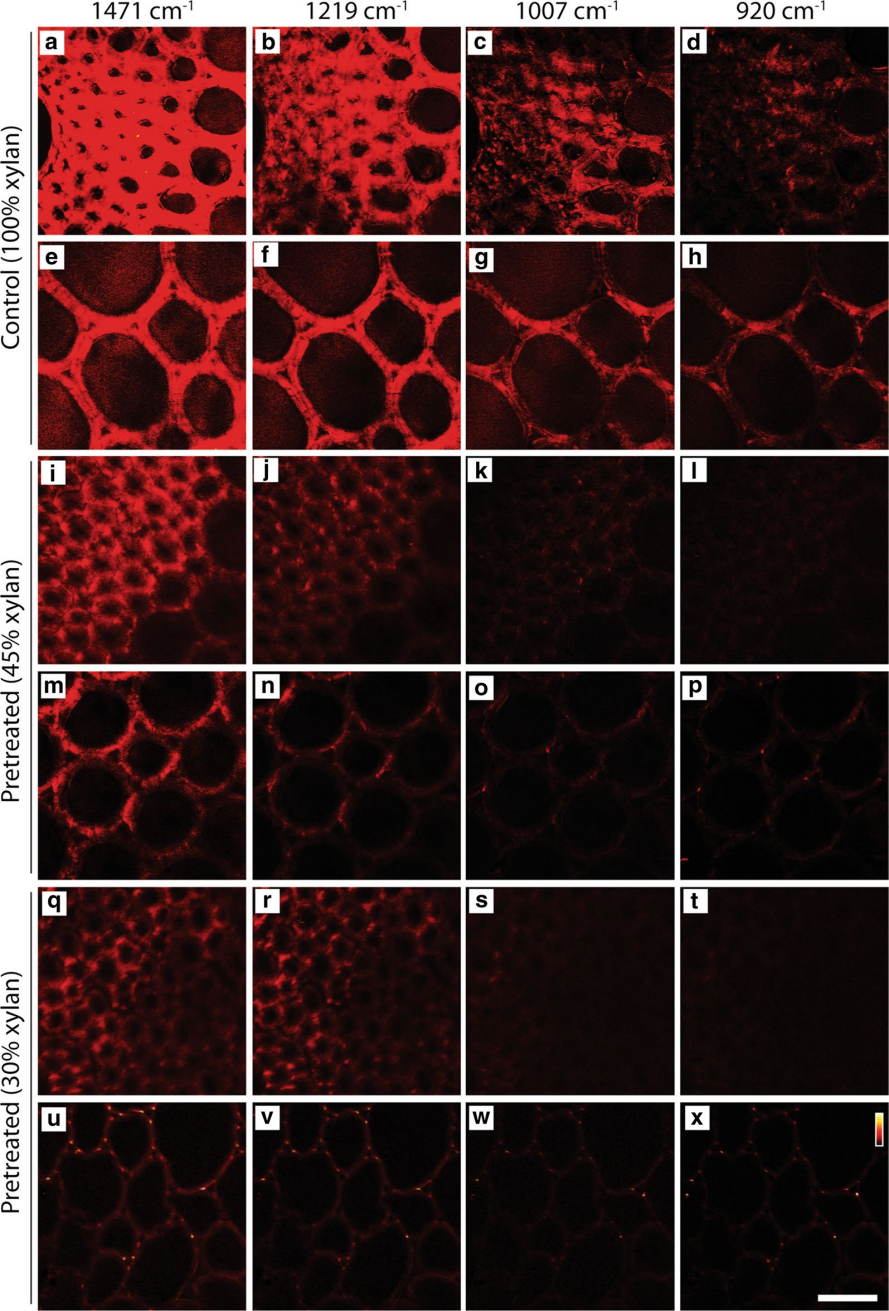
Comparison of SRS images of xylan in the native, untreated corn stover (control) and organosolv-pretreated corn stover cell walls. Two types of cell walls are compared: secondary cell walls in the vascular bundle (**a–d**, **i–l**, **q–t**) and parenchyma cell walls (**e–h**, **m–p**, **u–x**). *Scale bar* = 30 µm

The four xylan-specific Raman bands were also validated by SRS microscopy and tested for the possibility of in situ imaging of xylan in chemically pretreated plant cell walls. Native, untreated corn stover cell wall samples were organosolv-pretreated to remove 55–70% of the cell wall xylan (Additional file 1: Table S3, S4). Figure 5 compares the SRS images of two different types of cell walls, i.e., the vascular bundle cell wall and the parenchyma cell wall of the control and the xylan-reduced cell wall sample. When the relative cell wall xylan content was reduced from 100% to ~30 to 45% in the organosolv-pretreated cell walls, SRS signals from all four xylan frequencies also dropped. This correlation confirms that those Raman frequencies are specific to xylan content. Figure 5 also shows that the 1471 and 1219 cm^−1^ bands appear to have stronger SRS signals. Therefore, the SRS bands at 1471 and 1219 cm^−1^, especially the band at 1471 cm^−1^, might be more useful for imaging cell wall chemical details.

### In situ tracking of cell wall xylan during enzymatic digestion

**Fig. 6:**
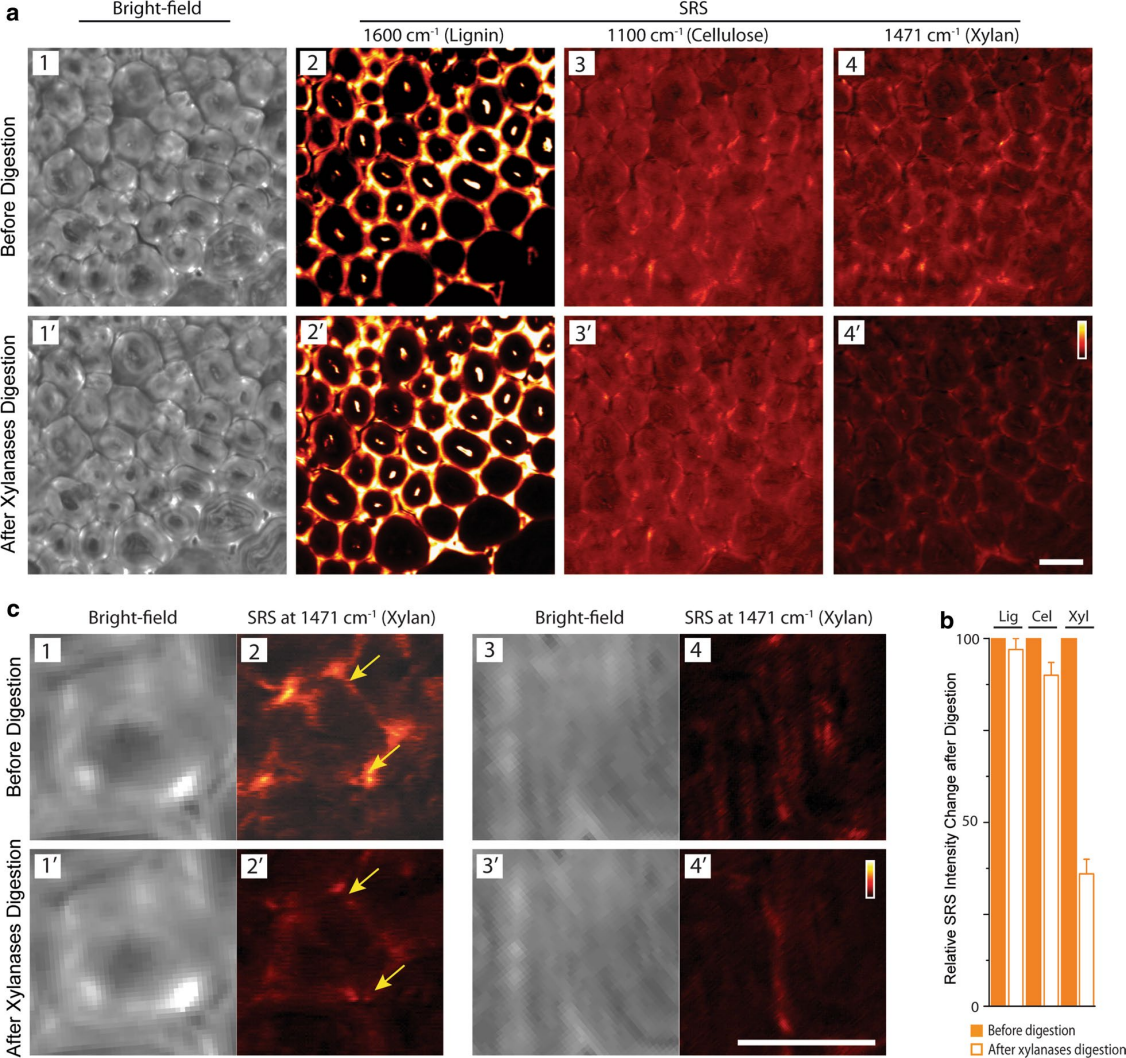
In situ tracking of lignin, cellulose, and xylan in deacetylated corn stover cell walls before and after xylan digestion by SRS. **a** Comparison of bright-field images of cell walls and SRS images of lignin, cellulose and xylan in the same cell walls. **b** Comparison of the relative overall SRS signal change in the images (before enzyme digestion = 100%) (*Lig* lignin, *Cel* cellulose and *Xyl* xylan; *error bar* are from 5 repeat digestion experiments). Lignin and cellulose are not affected by xylanases, and xylan is significantly reduced due to xylanases digestion. **c** Zoom-in bright-field images of cell wall and SRS images of xylan in two areas in vascular bundle region (**c** *1*–*4*: before xylan digestion; and **c** *1*′–*4*′: after xylan digestion) show significant xylan distribution changes in the cell wall due to the heterogeneous enzymatic digestion. Raman frequencies used for SRS imaging: lignin—1600 cm^−1^, cellulose—1100 cm^−1^ and xylan—1471 cm^−1^. *Scale bar* = 20 µm

We demonstrated the capability of SRS to in situ image xylan in cell walls. Xylan, cellulose, and lignin were imaged by SRS for the same cell wall region following xylanase digestion. Figure 6 shows the distribution of lignin (1600 cm^−1^), cellulose (1100 cm^−1^), and xylan (1471 cm^−1^) in the same cell walls, before and after xylanase treatment. As shown in bright-field images, xylan digestion did not introduce significant cell wall damage (Fig. 6a1, 1′). Lignin distribution was not affected by the xylanase digestion as can be seen in both of the lignin distribution images (Fig. 6a2, 2′); as well as in the comparison of overall image intensities (Fig. 6b) which shows almost no change before and after xylanase digestion. Cellulose Raman images show only a slight change (Fig. 6a3, 3′). In contrast to lignin and cellulose Raman channels, dramatic intensity drop was observed in the xylan channel (Fig. 6a4, 4′). Importantly, besides significant reduction in the SRS signal, xylan distribution in cell walls was also altered by enzymatic digestion, often taking on punctate type morphology (arrow, Fig. 6). Zoomed-in xylan images of two representative areas in the vascular bundle region show significant xylan distribution changes due to the enzymatic digestion.

**Fig. 7:**
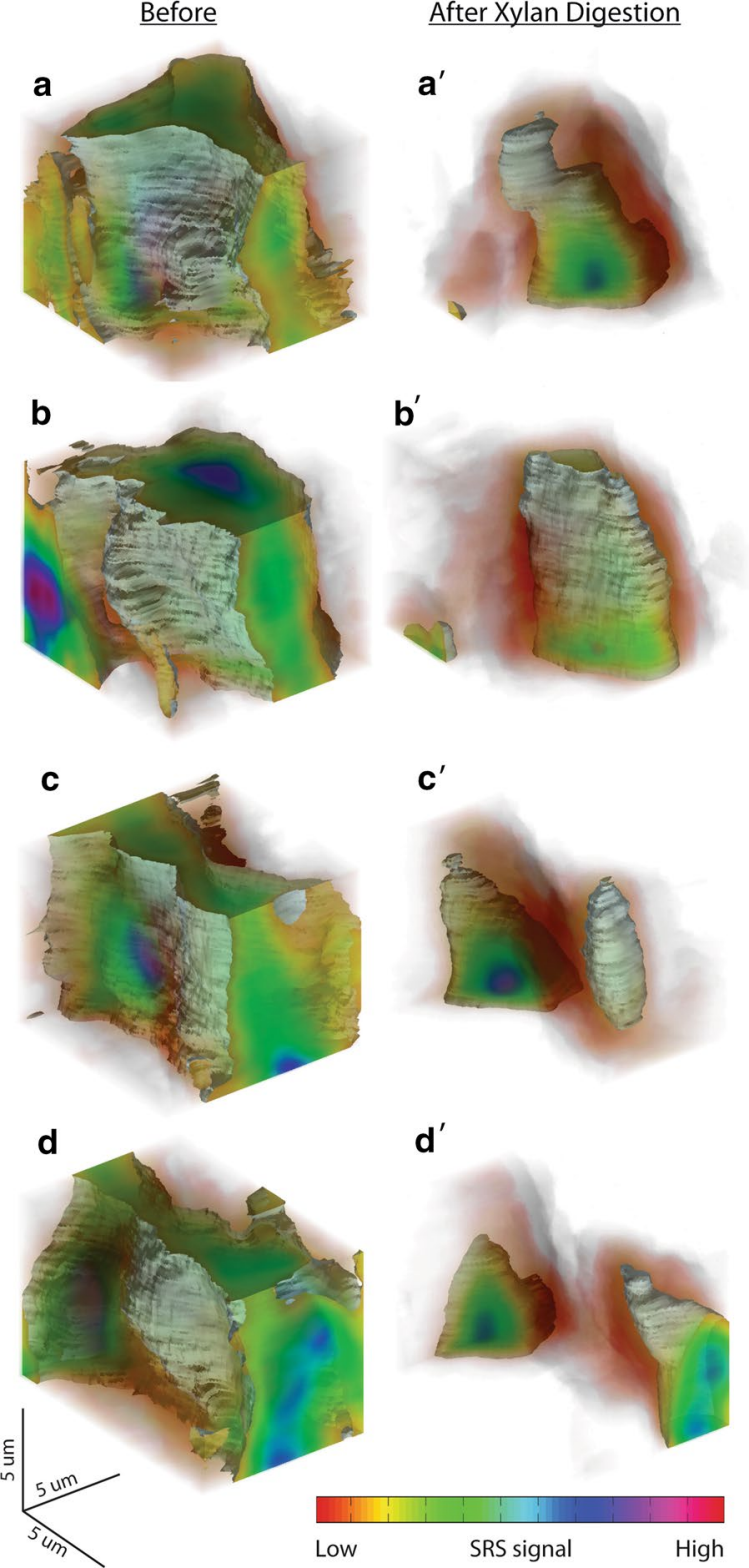
In situ 3D SRS imaging to track xylan distribution in deacetylated corn stover cell wall before and after xylan digestion. **a–d** Before digestion. **a′–d′** The same cell wall regions in **a**–**d** after xylanases digestion for comparison. Xylan Raman frequency at 1471 cm^−1^ was chosen for SRS imaging. **a, b, a′, b′** cell corner; **c, d, c′, d′**cell wall between two cell corners

More striking xylanases-induced change in xylan distribution can be revealed by the in situ 3D SRS imaging of xylan in the same cell wall region following xylanases digestion. Figure 7 compares the 3D reconstructions of xylan’s SRS signal at 1471 cm^−1^ before and after xylanases digestion. Xylan concentration as reflected by its SRS signal intensity clearly diminishes from cell lumen towards cell corner, whereas the remaining xylan is more located at cell corners. This 3D imaging technique could be useful for tracking xylan change under more complicate chemical/biological treatment conditions.

## Conclusions

We report the identification of xylan-specific Raman bands using xylanases to specifically remove xylan from the polymer matrix of deacetylated corn stover cell walls. We further used SRS microscopic imaging to verify these xylan-specific Raman band assignments using organosolv-pretreated native corn stover, which confirmed that these Raman bands are closely associated with the xylan content. Using the highest sensitivity xylan band as an example, we demonstrated that xylan can be resolved from cellulose and lignin in situ using enzymatic digestion and label-free SRS microscopy in both 2D and 3D. We believe that this novel approach can be used to map xylan in plant cell walls and that this ability will enhance our understanding of the role played by xylan in cell wall biosynthesis and deconstruction.

## Methods

### Deacetylated corn stover

The control corn stover was deacetylated and disc refined as previously described [63], to remove approximately 80% of the acetyl groups and improve xylanase digestion without significantly altering the cellulose, xylan, and lignin content. In brief, dry native corn stover was added to a dilute 0.1 M sodium hydroxide to make the final 8% (w/w) total solids slurry. The slurry was heated to 80 °C for 2 h, then drained, extensively washed and dried using a continuous screw press. The solids fraction was further milled with a commercial scale disc refining facility at the Andritz pilot plant and R&D laboratory in Springfield (Ohio, United States).

Deacetylated corn stover sections were prepared by deacetylation of corn stover rind sticks (~1 cm long) using 0.1 M hydroxide at 75 °C. Deacetylated rind sticks were rinsed extensively until pH ~7 was attained and then transversely sectioned into 50-µm slices with a rotary microtome (RM2235, Leica).

### Xylan model compounds

Oat spelts xylan (X-0627), birchwood xylan (X-0502), and d-xylose (58-86-6, >99%) were from Sigma-Aldrich, Missouri, United States. Xylo-oligosaccharides with degree of polymerization ranging from two to seven were from Cascade Analytical Reagents & Biochemical, CAS 9014-63-5. The chemical compositional analysis for xylan model compounds was done by NREL laboratory analytical procedures [64]. Basically, a strong sulfuric acid solution was used for primary hydrolysis of the sample, followed by dilution with water and a secondary high-temperature hydrolysis step. This procedure hydrolyzes the carbohydrate fraction to soluble monosaccharides, leaving a lignin-rich residue to be vacuum filtered and measured gravimetrically. The sugars in the hydrolysate solution were measured as monomers to determine the carbohydrate fraction of the sample. Spelts xylan also contains 3–4% glucuronic acids as part of xylan aside from the above NREL chemical compositional results. Birchwood xylan contains about 10–12% glucuronic acid as part of xylan.

### Enzymatic digestion of deacetylated corn stover to partially remove xylan from cell walls

The deacetylated corn stover solids were washed with 20 mM sodium acetate and 100 mM sodium chloride buffer (pH 4.8) solution. The commercial enzyme product (Multifect^®^ Xylanase from DuPont™ Genencor^®^, Palo Alto, California, United States) was desalted in 10 mL aliquots using two serial HiPrep 26/10 desalting columns (GE Life Sciences, Piscataway NJ) equilibrated in 20 mM sodium acetate buffer (pH 4.8) with 100 mM sodium chloride. Protein containing fractions were pooled and protein concentration determined using the BCA protein assay (Pierce Rockford, IL). Enzyme samples were desalted less than 2 days before use, with fresh material being generated for each experiment as desalted commercial enzymes tend to degrade and precipitate within a few days. The enzyme solution was added to same buffer solution containing deacetylated corn stover solids at 1% with enzyme loading at 3.6–58 mg/g xylan. Enzymatic digestions were performed in 2.5 mL shaker tubes in a shaking incubator at 45 °C and 150 rpm for up to 40 h.

### Organosolv treatment of native corn stover

Organosolv treatment of native corn stover was performed following the same experimental procedure published previously [65]. The solution for organosolv treatment contained methyl isobutyl ketone (MIBK), acetone and water in the following ratio: MIBK:acetone:water = 11:44:44 (g:g:g, 100 mL). The sulfuric acid concentration remained at 1.2 wt%. After 2 min at 160 °C, 55% of the cell wall xylan was solubilized (45% remaining). After 10 min, 70% of cell wall xylan was solubilized (30% remaining). The xylan removal was measured according to the same experimental procedure published previously [65]. Basically, it was calculated by comparing the total amount of xylan (from compositional analysis of sample prior treatment) and the amount of sugar monomers in supernatant after organosolv treatment.

### Raman spectroscopic analysis

All Raman spectra were acquired on a LabRam HR800 confocal Raman system (Horiba Jobin–Yvon, Edison, New Jersey, United States) equipped with an Olympus BX41 microscope and a 785 nm diode laser source. Sample powder was placed on a glass cover slide for measurement. The excitation beam was focused above the sample through a 40X objective lens (Olympus, UPlanSApo, 0.95 NA). The Raman scattering light from the sample was collected by the same objective lens. Before each spectrum acquisition, the sample was photo-bleached for 30 min to effectively reduce the fluorescence background. This is indicated as the baseline shift between two adjacent spectrum scans, which was less than 10%. With fluorescence background reduced, long integration times (usually 200 s) were used so that the maximum Raman signal intensity is about ~80% of the full dynamic range of the CCD detector. For each sample, more than 30 times accumulations were acquired to enhance the signal-to-noise ratio. Since in confocal Raman microscopy, the sampling volume is small and to minimize sampling error, more than 30 samples were measured for the each condition.

### SRS imaging

The SRS imaging was conducted on the same SRS microscope as described previously [25]. In brief, a high-power Nd:YO4 oscillator (HighQ picoTRAIN, Spectra-Physics) producing 7 ps pulse trains at 1064 nm (15 W max) and 532 nm (9 W max) was used. 2 W of the 1064 nm light was used as the Stokes beam. The 532 nm beam was directed to pump an optic parametric oscillator (Levante Emerald, APE GmbH, Germany) to produce 6 ps tunable wavelength pulse train as the pump beam. The wavelength of the pump beam was adjusted for the selected Raman frequency. For example, it was tuned to 909.2 nm for lignin’s resonance frequency at 1600 cm^−1^. The Stokes beam was intensity-modulated by an acoustic optic modulator (3080-122, Crystal Technology) at 10 MHz with 80% modulation depth, and then combined with the pump beams by a long-pass beam combiner (1064dcrb, Chroma). The two beams were routed to a custom modified mirror-scanning microscope system (BX62WI/FV300, Olympus) attached with an Olympus inverted microscope. Typical laser power at the sample plane was 80 mW for each beam, and this allowed for continuous imaging without causing any noticeable photo-damage. The light transmitted through the sample was collected by a high numeric aperture condenser (1.45 NA O, Nikon), and filtered by an optical filter (CARS980/220, Chroma) to block the Stokes beam completely so that only amplitude modulation on the pump beams due to the SRS process was detected. The pump beam intensity was detected by a large-area silicon PIN photodiode (FDS1010, Thorlabs) back-biased at 70 V. A lock-in amplifier (SR844, Stanford Research Systems) was used to detect the intensity change in the pump beam. 3D SRS imaging was performed by collect a stack of images along Z axis and the 3D rendering of the image stack was processed in Matlab.

### Xylanase digestion of deacetylated corn stover transverse sections

An incubation chamber was constructed by two pieces of #1 glass microscope coverslips sandwiched by a silicon separator (Grace Bio-Labs, Oregon, United States). One slice of the 50-μm deacetylated corn stover slice was immersed in 125 µL 20 mM sodium acetate and 100 mM sodium chloride buffer (pH 4.8) solution containing Multifect^®^ Xylanase (DuPont™ Genencor^®^, Palo Alto, California, United States). The enzyme loading was approximately 93 mg/g xylan. The chamber was incubated at 45 °C for 40 h for enzymatic digestion.

## Additional file

**Additional file 1: Figure S1.** Basic chemical units of cellulose and xylan, and the two most common ordered cellulose structures. **Table S1.** Chemical compositional analysis of sugar content in the supernatant from the xylanase digested deacetylated corn stover cell wall. **Table S2.** Extent of sugars hydrolyzed from xylanase digestion of deacetylated corn stover. **Table S3.** Chemical compositional analysis of the supernatants from organosolv pretreated corn stover (compound per biomass). **Table S4.** Percentage of xylose and lignin dissolved from organosolv pretreated corn stover. **Figure S2.** SRS images of lignin, cellulose and xylan distribution in deacetylated and disc refined corn stover fragments before and after cell wall xylan removal by xylanases. ESI References.

## Abbreviations

SRS: stimulated Raman scattering
CARS: coherent anti-stokes Raman scattering
