# Supplementary material for: In situ label-free imaging of hemicellulose in plant cell walls using stimulated Raman scattering microscopy
Source: Biotechnol Biofuels. 2016 Nov 22;9:256. doi: 10.1186/s13068-016-0669-9 (PMC5120481; doi:10.1186/s13068-016-0669-9)
Supplement: Supplementary file 1 — Additional file 1: Figure S1. Basic chemical units of cellulose and xylan, and the two most common ordered cellulose structures. Table S1. Chemical compositional analysis of sugar content in the supernatant from the xylanase digested deacetylated corn stover cell wall. Table S2. Extent of sugars hydrolyzed from xylanase digestion of deacetylated corn stover. Table S3. Chemical compositional analysis of the supernatants from organosolv pretreated corn stover (compound per biomass). Table S4. Percentage of xylose and lignin dissolved from organosolv pretreated corn stover. Figure S2. SRS images of lignin, cellulose and xylan distribution in deacetylated and disc refined corn stover fragments before and after cell wall xylan removal by xylanases. ESI References. [file 13068_2016_669_MOESM1_ESM.docx]

Supplementary Information for:

***In Situ* Label-free Imaging of Hemicellulose in Plant Cell Walls**

**Using Stimulated Raman Scattering Microscopy**

Yining Zeng^1,3*^, John M. Yarbrough^1,3^, Ashutosh Mittal^1^, Melvin P. Tucker^2,3^, Todd B. Vinzant^1^, Stephen R. Decker^1,3^ and Michael E. Himmel^1,3*^

^1^Biosciences Center, ^2^National Bioenergy Center, National Renewable Energy Laboratory, Golden, CO 80401 and ^3^BioEnergy Science Center (BESC), Oak Ridge National Laboratory, PO Box 2008 MS6341, Oak Ridge, TN 37831

*Corresponding authors: Yining.Zeng@nrel.gov and Mike.Himmel@nrel.gov

Contents

[Figure S1. Basic chemical units of cellulose and xylan, and the two most common ordered cellulose structures 2](#_Toc466029207)

[Table S1. Chemical compositional analysis of sugar content in the supernatant from the xylanase digested deacetylated corn stover cell wall. 3](#_Toc466029208)

[Table S2. Extent of sugars hydrolyzed from xylanase digestion of deacetylated corn stover. 3](#_Toc466029209)

[Table S3. Chemical compositional analysis of the supernatants from organosolv pretreated corn stover (compound per biomass) 4](#_Toc466029210)

[Table S4. Percentage of xylose and lignin dissolved from organosolv pretreated corn stover 4](#_Toc466029211)

[Figure S2. SRS images of lignin, cellulose and xylan distribution in deacetylated and disc refined corn stover fragments before and after cell wall xylan removal by xylanases 5](#_Toc466029212)

[ESI References 6](#_Toc466029213)


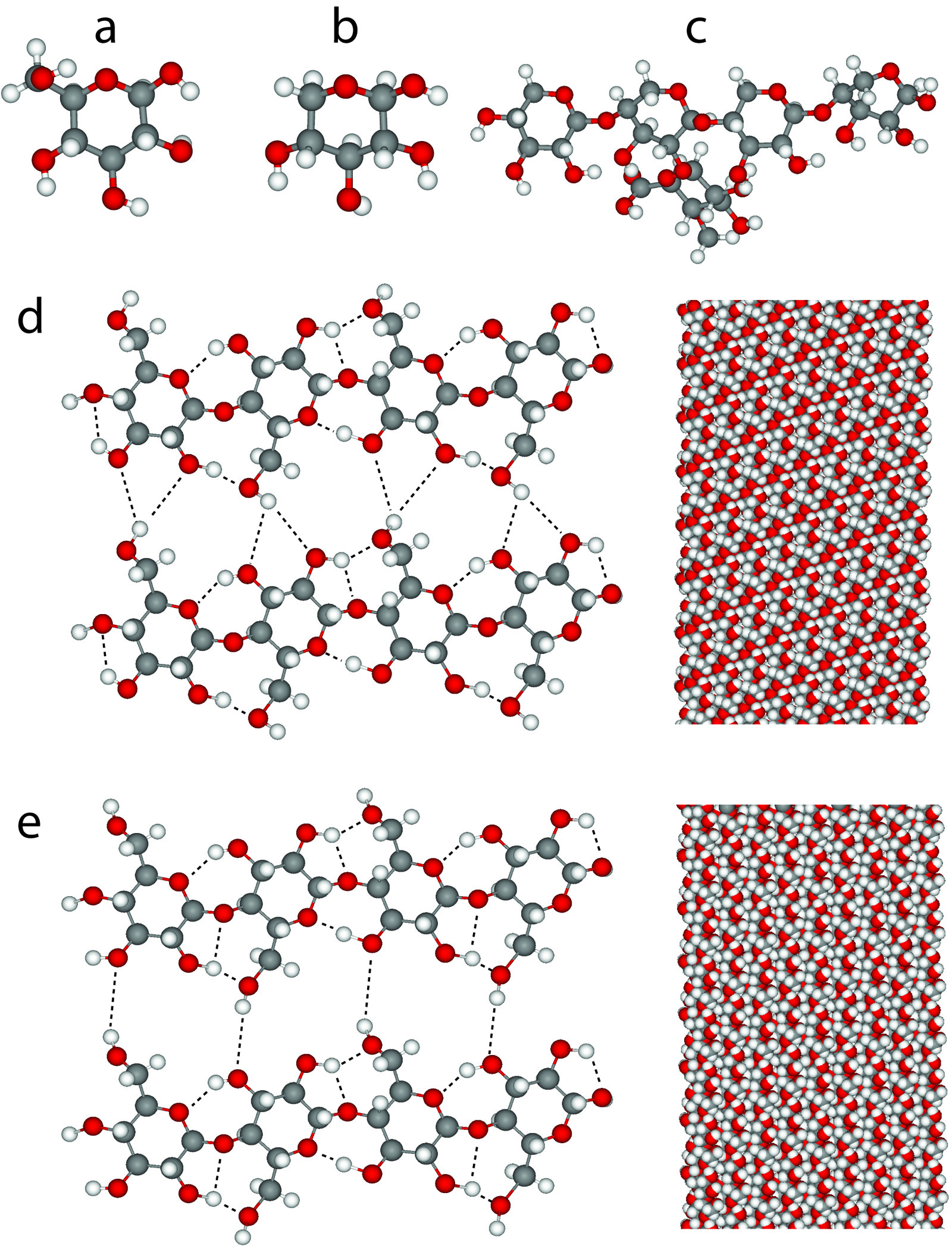


Figure S1. Basic chemical units of cellulose and xylan, and the two most common ordered cellulose structures. **(a)** Chemical structure of glucose. **(b)** Chemical structure of xylose. **(c)** Chemical structure of 4-*O*-methyl glucuronoxylan, a common unit for xylan backbone with branching sidechains which prohibit xylan forming ordered structures as found in cellulose. **(d)** I*a* and **(e)** I*b* hydrogen bond structures based on the crystal structures of Nishiyama et al.[[1](#_ENREF_1), [2](#_ENREF_2)]; and the side-views of the resulting ordered cellulose microfiber structures: carbon atom – grey; oxygen atom – red; hydrogen atom - white. Hydrogen bonds are represented by dotted lines.

# Table S1. Chemical compositional analysis of sugar content in the supernatant from the xylanase digested deacetylated corn stover cell wall.

| concentration  g/L  Enzyme  loading  mg/g xylan | Cellobiose | Glucose | Xylose | Arabinose | Xylitol | Lactic acid | Glycerol | Acetic acid* |
| --- | --- | --- | --- | --- | --- | --- | --- | --- |
| 3.6 | 0.00 | 0.00 | 1.02 | 0.00 | 0.00 | 0.00 | 0.00 | 0.59 |
| 3.6 | 0.00 | 0.00 | 0.93 | 0.00 | 0.00 | 0.00 | 0.00 | 0.60 |
| 7.2 | 0.00 | 0.00 | 1.31 | 0.00 | 0.00 | 0.00 | 0.00 | 0.60 |
| 7.2 | 0.00 | 0.00 | 1.43 | 0.00 | 0.00 | 0.00 | 0.00 | 0.60 |
| 14 | 0.00 | 0.00 | 1.80 | 0.00 | 0.00 | 0.00 | 0.00 | 0.60 |
| 14 | 0.00 | 0.00 | 1.58 | 0.00 | 0.00 | 0.00 | 0.00 | 0.60 |
| 29 | 0.00 | 0.00 | 2.25 | 0.00 | 0.00 | 0.00 | 0.00 | 0.60 |
| 29 | 0.00 | 0.00 | 1.88 | 0.00 | 0.00 | 0.00 | 0.00 | 0.60 |
| 58 | 0.00 | 0.00 | 2.02 | 0.00 | 0.00 | 0.00 | 0.00 | 0.60 |
| 58 | 0.00 | 0.01 | 2.07 | 0.00 | 0.00 | 0.00 | 0.00 | 0.60 |

*Acetic acid presents in the buffer solution.

#

# Table S2. **Extent of sugars hydrolyzed from xylanase digestion of deacetylated corn stover.**

| Faction hydrolyzed  Enzyme loading  mg/g xylan | Concentration (mg/g) | Xylan | Glucose |
| --- | --- | --- | --- |
| 3.6 mg/ml | 3.6 | 25±1.4% | 0 |
| 7.2 mg/ml | 7.2 | 36±1.8% | 0 |
| 14 mg/ml | 14 | 44±3.8% | 0 |
| 29 mg/ml | 29 | 55±8.2% | 0 |
| 58 mg/ml | 58 | 55±0.6% | 0 |

Table S3. Chemical compositional analysis of the supernatants from organosolv pretreated corn stover (compound per biomass).

| Percentage in Biomass  Pretreatment  Conditions | Arabinose | Galactose | Glucose | Xylose | Lignin |
| --- | --- | --- | --- | --- | --- |
| 160⁰C, 2 min | 0.6% | 0.2% | 2.3% | 9.2% | 14.1% |
| 160⁰C, 10 min | 0.8% | 0.3% | 3.1% | 12.5% | 22.8% |

Table S4. Percentage of xylose and lignin dissolved from organosolv pretreated corn stover.

| Fraction of  Dissolved  Pretreatment  Conditions | Xylose | Lignin |
| --- | --- | --- |
| 160⁰C, 2 min | 55% | 61% |
| 160⁰C, 10 min | 70% | 94% |


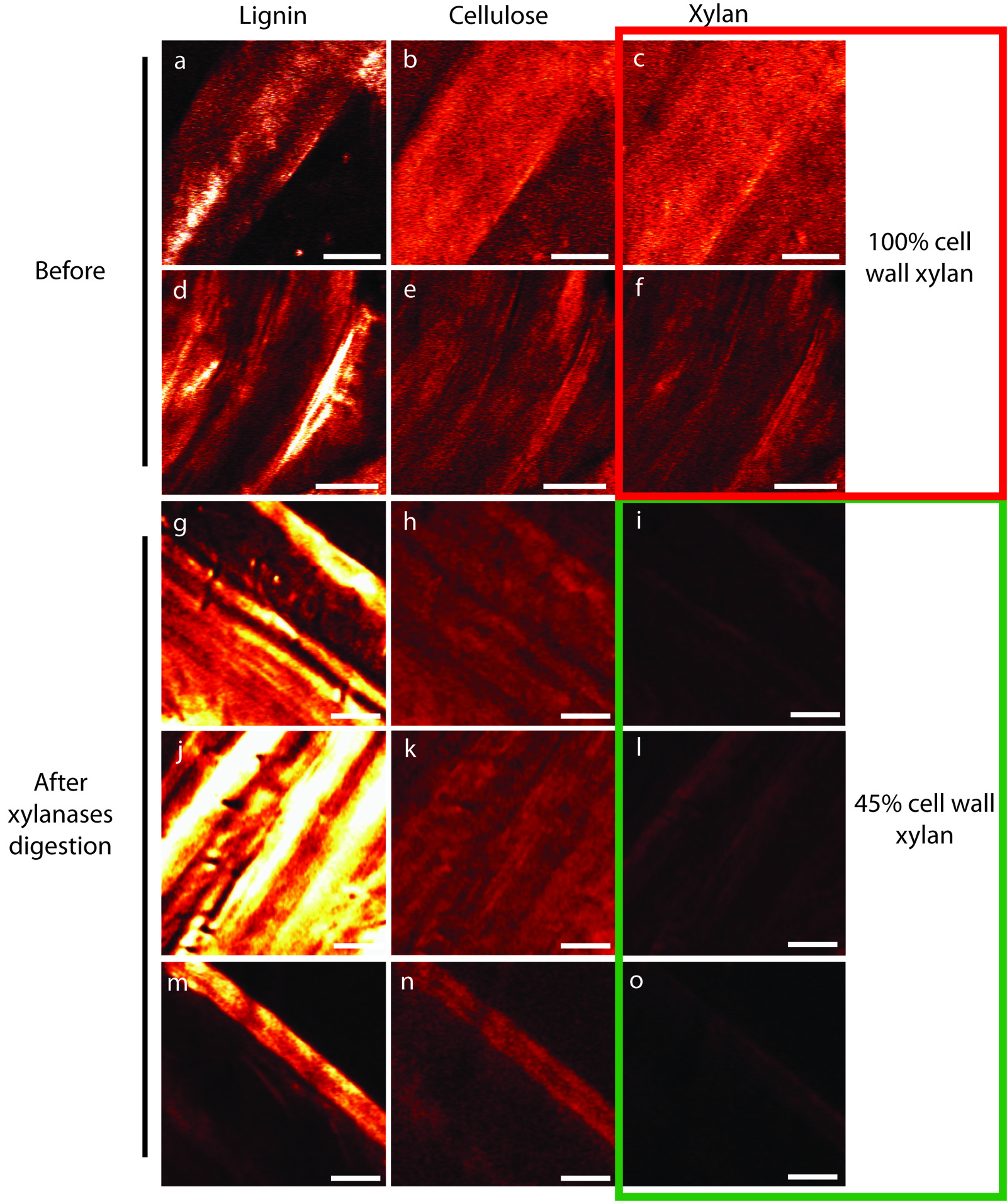


Figure S2. SRS images of lignin, cellulose and xylan distribution in deacetylated and disc refined corn stover fragments before and after cell wall xylan removal by xylanases**. (a**-**f)** Before digestion. **(g-o)** After Multifect® Xylanase digestion. Enzyme loading was 58 mg/g xylan at 45ºC for 40 h. 55% of the cell wall xylan was removed by digestion. The lignin SRS signal from 1600 cm^-1^(**a**, **d**, **g**, **j** and **m**) and cellulose SRS signal from 1100 cm^-1^ (**b**, **e**, **h**, **k**, **n**) were not affected by xylan removal. Xylan SRS signal (1219 cm^-1^) was drastically reduced (**c**, **f** versus i, l, o) due to xylan solubilization by xylanases. Scale bar = 10 µm.

# ESI References

1. Nishiyama Y, Langan P, Chanzy H. Crystal Structure and Hydrogen-Bonding System in Cellulose Iβ from Synchrotron X-ray and Neutron Fiber Diffraction. Journal of the American Chemical Society. 2002;124(31):9074-82. doi:10.1021/ja0257319.

2. Nishiyama Y, Sugiyama J, Chanzy H, Langan P. Crystal Structure and Hydrogen Bonding System in Cellulose Iα from Synchrotron X-ray and Neutron Fiber Diffraction. Journal of the American Chemical Society. 2003;125(47):14300-6. doi:10.1021/ja037055w.
